# S238. DEVELOPMENT OF SELF-STIGMA INVENTORY FOR THE RELATIVES OF THE PATIENTS WITH SCHIZOPHRENIA: RELIABILITY AND VALIDITY STUDY IN TURKEY

**DOI:** 10.1093/schbul/sby018.1025

**Published:** 2018-04-01

**Authors:** Mustafa Yildiz, Aysel Incedere, Fatma Kiras, Fatma Betül Abut, Ayşe Kircali, Kübra Ipçi

**Affiliations:** Kocaeli University

## Abstract

**Background:**

Stigmatization refers to a disrespectful attribution to a person account of being considered as outside of society’s norms. Stigmatization happens not only towards the mentally ill patients but can also occur against the relatives of the patients. Researches revealed that relatives of the patients hide this disease from other people, ashamed of their patients, and feel excluded from others. They may choose to conceal the disease or even the name of the illness. This situation is referred as self-stigma of the relatives. It is important to develop an instrument to evaluate and assess the self-stigma of the relatives since it increases their burden and negatively affects the prognosis of the illness. The purpose of this study was to develop a culturally-sensitive and user-friendly inventory for the assessment of self-stigma of families.

**Methods:**

After examining the studies that investigate self-stigma and internalized stigma in people with mental illness, 25-item inventory was formed. Focus group interviews were conducted with a sample of 18 relatives of the patients with schizophrenia, and the items were reviewed and rephrased into more comprehensible and relevant statements for the relatives. Consequently, the inventory was finalized with 19 items. A pilot study was carried out with 15 relatives, and the inventory was reevaluated in terms of its comprehensibility and usability. One hundred and six relatives of the patients with schizophrenia and schizoaffective disorder were given a sociodemographic form, Self-Stigma Inventory for Relatives (SSI-R), Beck Depression Inventory (BDI), Beck Hopelessness Scale (BHS), Rosenberg Self-Esteem Scale (RSES), and Zarit Caregiver Burden Scale (ZCBS). For reliability analyses; internal consistency coefficient, item-total correlation, and split-half reliability were calculated. For validity analyses; explanatory factor analysis and convergent validity were assessed.

**Results:**

The sample was consisted of 106 relatives whose 52% were female, 77% were married, and the level of education was 9 years.

Cronbach’s alpha coefficient for SSI-R total score was calculated as 0.87, while Cronbach’s alpha scores for subscales were found between 0.82 and 0.83. Split-half reliability coefficient was 0.79. For factor analysis, Kaiser-Meyer-Olkin value was found as 0.801 and Barlett test was significant (p<0.001). In explanatory factor analysis, 5 factors were detected whose eigenvalue was greater than one and the factors could explain 73% of the total variance. When the scree plot was examined, it was observed that the slope suddenly decreased and changed after the third factor. For this reason, varimax rotation was applied with three factors. Consequently, 3 factors (perceived incompetency, internalized stereotypes and social withdrawal, concealment of the illness) were detected and they could explain 66% of the total variance. Five items were omitted since they had lower factor value than 0.40. On the last form, first factor included 6 items, second factor had 5 items, and third factor had 3 items.

SSI-R was correlated with Beck Depression Inventory (r=0.20, p<0.05), Beck Hopelessness Scale (r=0.19, p<0.05), Zarit Caregiver Burden Scale (r=0.41, p<0.001), and Rosenberg Self-Esteem Scale (r=-0.23, p<0.05).

**Discussion:**

This study shows that the SSI-R is a reliable and valid instrument on assessing the stigmatization in families. The scale is easy-to-use with its 14 items. It can be considered as a valuable instrument to use for research and therapeutic purposes.

